# Influence of Angle *κ* and Higher-Order Aberrations on Visual Quality Employing Two Diffractive Trifocal IOLs

**DOI:** 10.1155/2019/7018937

**Published:** 2019-11-27

**Authors:** Cecilio Velasco-Barona, Claudia Corredor-Ortega, Alejandra Mendez-Leon, Nadia L. Casillas-Chavarín, Daniel Valdepeña-López Velarde, Guadalupe Cervantes-Coste, Daniel Malacara-Hernández, Roberto Gonzalez-Salinas

**Affiliations:** ^1^Anterior Segment Surgery Department, Asociación para Evitar la Ceguera, Mexico City 04030, Mexico; ^2^Centro de Investigaciones en Óptica A.C. (CIO), León 37150, Guanajuato, Mexico; ^3^Laboratorio Nacional de óptica de la Visión, León, Mexico; ^4^Research Department, Asociación para Evitar la Ceguera, México City 04030, Mexico

## Abstract

Prospective, randomized, comparative, and controlled study to estimate the association between angle *κ* distance and higher-order aberrations (HOAs) with postoperative visual acuity after presbyopia-correcting IOL implantation. Forty-three eyes from 43 patients were included and randomly assigned in two groups for either AT LISA tri 839MP or Acrysof IQ PanOptix IOL implantation. The OPD-Scan III analyzer was utilized to assess the angle *κ* distance and higher-order aberration (HOAs). Twenty-three eyes were in the Acrysof IQ PanOptix group and 20 patients in the AT LISA tri 839MP group. The uncorrected distance visual acuity (UDVA) for the PanOptix group was 0.092 ± 0.10, whereas for AT LISA tri was 0.050 ± 0.06 (*P*=0.229). The uncorrected intermediate visual acuity (UIVA) for the PanOptix group was 0.173 ± 0.18, whereas for AT LISA tri, it was 0.182 ± 0.11 (*P*=0.669). Uncorrected near visual acuity (UNVA) was 0.068 ± 0.04 and 0.085 ± 0.07, respectively (*P*=0.221). Also, correlation coefficient between HOAs and the Strehl ratio for each group were −0.768 (*P* < 0.0001) and −0.863 (*P*=0.0001). Patients implanted with both trifocal IOLs showed excellent postoperative visual performance at all distances at the six-month follow-up visit. No association was found between angle *κ* distance and postoperative visual acuity regardless of the angle *κ* magnitude or the two trifocal IOLs inner optical diameter. Also, internal aberrations demonstrated a significant inverse correlation with the Strehl ratio for both trifocal IOLs.

## 1. Introduction

Trifocal intraocular lens implantation has become an ever more common solution for cataract patients who pursuit a spectacle-free option after IOL surgery [1]. Surgical planning, therefore, poses a significant challenge to achieve spectacle independence and at the same time meet visual expectations [1, 2].

Preoperative assessment should be aware among others of pupil size, preoperative angle *κ*, and significant astigmatism as key variables that may affect the perceived outcome for patients who have a presbyopia-correcting IOL [3]. In addition, tilt and decentration could inflict a negative impact on the eye's optical performance, inducing asymmetric aberrations that in severe cases can decrease optical quality [4, 5].

Also, a functional deviation between the optical center, the visual axis, and the pupillary axis of the multifocal IOL can lead to higher-order aberrations postoperatively, resulting in decreased visual quality. Therefore, some propose including the measurement of angle kappa (*κ*) and angle alpha (*α*) in preoperative examinations of eyes scheduled for multifocal IOL implantation. Although recognition of the importance of angle *κ* and angle *α* for successful multifocal IOL implantation is growing, there are little data regarding their impact on objective visual quality.

**Figure 1:**
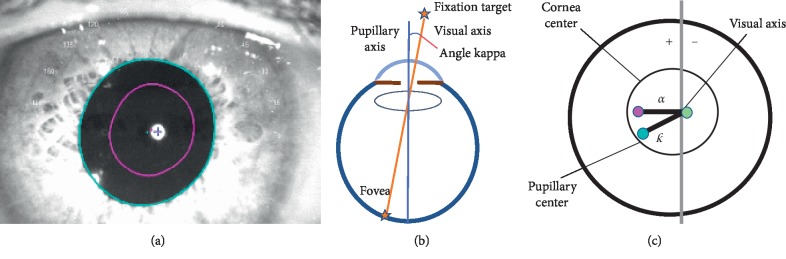
Pupillary diameter display and diagram of *κ* angle distance. (a) Comparison of pupillary diameter under mesopic and photopic conditions. (b) Diagram of *κ* angle formed by the visual axis and the pupillary axis. (c) Graphic representation of *κ* angle, visual axis, and pupillary axis, showing the center of the visual axis (green cross, representing the center of the reflection points), corneal center (violet dot in diagram, similar to the anatomic center), and pupillary center (blue dot, representing the center of the circle). The radial distance between the green cross and the violet dot represents angle alpha (*α*). The radial distance between the blue dot and the green cross represents angle *κ* (*κ*). The + sign represents the positive angle; and the—sign represents the negative angle.

In cases with a considerable angle *κ*, there is a greater chance of a decentration due to the increased distance between the pupillary light reflex and corneal light reflex, as depicted in Figure 1, which could lead to functional decentration of the trifocal IOL [6].

Most presbyopia-correcting IOLs have multiple concentric rings in them with varying powers, and therefore a mild IOL decentration could result in decreased vision, inducing high-order aberrations and photic phenomena including decreased contrast sensitivity, glare, and halos [6–8]. Although acknowledgment of the importance of angle *κ* for successful multifocal IOL implantation is increasing, few data regarding their impact on objective visual quality is widely available.

This study outlines the overall associations between angle *κ* distance and both the total and the internal HOAs when using two trifocal IOLs. It has been described that the optical axis/center of the capsular bag may not match the patient's visual axis when a considerable angle *κ* distance (>0.5 mm) is evidenced, leading to potentially poor outcomes when using a trifocal IOL [6].

The purpose of the present study was to estimate the association between angle *κ* distance and higher-order aberrations (HOAs) with postoperative visual acuity and vision quality after presbyopia-correcting IOL implantation employing either AT LISA tri 839MP or Acrysof IQ PanOptix IOL.

## 2. Materials and Methods

### 2.1. Design and Setting

This prospective, randomized, comparative and controlled study included patients undergoing Multifocal IOL surgery at the Anterior Segment Surgery Department at the Asociación para Evitar la Ceguera, Mexico City, Mexico. The Internal Review Board approved this study, which was conducted following the tenets of the Declaration of Helsinki and Good Clinical Practices Guidelines. All participants were briefed extensively and provided written informed consent before measurements were performed.

### 2.2. Patients

Cataract patients >50 years of age with lens opacities graded from NO1NC1 to NO3NC3 according to the LOCS III classification system undergoing routine phacoemulsification cataract extraction with trifocal IOL implantation were included [9]. Preoperative exclusion criteria for the study included corneal astigmatism over 1.0 D, ocular pathologies such as amblyopia, dry eye disease, evidence for corneal dystrophy, retinal pathology, glaucoma, and previous ocular surgery. The study comprised a total of 43 eyes from 43 patients: twenty-three eyes in the Acrysof IQ PanOptix group and twenty patients in the AT LISA tri 839MP group.

### 2.3. Experiment Design

**Figure 2:**
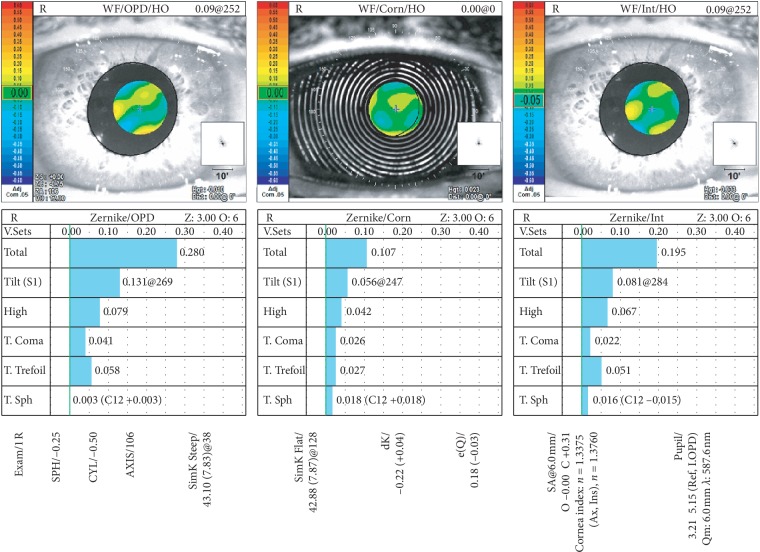
HOAs measurements using an OPD-Scan III analyzer obtained at the six-month follow-up visit.

Prior to the surgical procedure, partial coherence interferometry- (PCI-) based IOL calculation was obtained for all cases (IOLMaster 500, Carl Zeiss Meditec AG). Forty-six included patients were randomly assigned to two groups for either an AT LISA tri 839MP or an Acrysof IQ PanOptix IOL implantation after routine cataract removal (twenty-three patients per group). An OPD-Scan III analyzer (NIDEK CO., LTD., Tokyo, Japan) was utilized to assess both the angle *κ* distance, defined by the radial distance between the center of the pupil and the visual axis (see Figure 1), HOAs measurements, and the Strehl ratio for vision quality at the six-month follow-up visit, as depicted in Figure 2.

### 2.4. Instrumentation

#### 2.4.1. IOL Master 500

A noncontact optical biometer was employed; measuring the distance from the corneal vertex to the retinal pigmented epithelium (RPE): The IOL Master 500 (Carl Zeiss Meditec AG) measures the axial length, using PCI with a 780 nm laser diode infrared light. Also, keratometry, white to white distance, and anterior chamber dept, from the corneal epithelium to the anterior surface of the lens, were measured using image analysis. Each measurement requires the instrument to be aligned with the visual axis [10].

#### 2.4.2. OPD-Scan III Analyzer

An OPD-Scan III aberrometer provided the total and intraocular high-order aberration (HOA) data, including the Strehl ratio, with a mesopic pupil under mesopic (3 cd/m2) lighting conditions [11]. The OPD-Scan III provides a complete set of maps, including four different corneal topography maps, local refractive power of the entire eye due to aberrations at various locations within the pupil, a variety of wavefront aberration maps, and photopic and mesopic pupillometry. By computing the corneal wavefront aberration and comparing it with the total wavefront map, it is possible to estimate optical quality due to the internal aberrations of the eye. The internal aberrations represent all aberrations behind the anterior corneal surface. Wavefront data are gathered from available zones up to a 9.5 mm area including 2,520 data point analyses, in 7 zone measurement, adding the capability to provide for the calculation of mesopic refractions. Placido disc topography measures 33 rings in a vertical position and 39 in the horizontal position, including 11,880 data points [11].

### 2.5. Main Outcome Measure

Angle *κ* distance was assessed as the extrapolated distance that overlapped the center of the pupil and the corneal reflex. The total and internal aberrations were evaluated separately to differentiate aberrations originated from the total optic system from the internal aberrations of the eye.

Also, the uncorrected distance visual acuity (UDVA), uncorrected near visual acuity (UNVA), and uncorrected intermediate visual acuity (UIVA) were evaluated. Visual acuities were measured under photopic conditions using Snellen visual charts and then converted into logarithm of the minimum angle of resolution (logMAR) notation.

**Figure 3:**
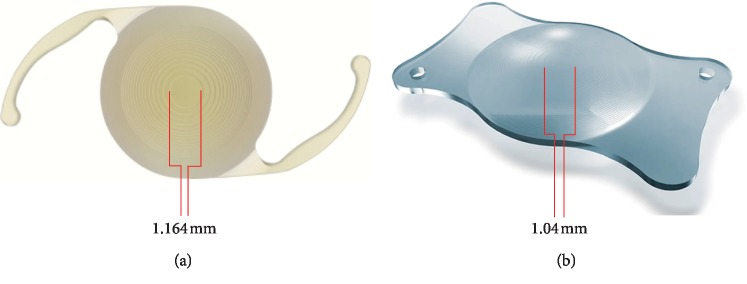
Inner ring optical diameter of the trifocal IOLs included in the study: (a) Acrysof IQ PanOptix® and (b) the AT LISA tri 839MP®.

Key optical and physical features of each IOL are summarized in Table 1. A depiction of both Trifocal IOLs is shown in Figure 3.

### 2.6. Surgical Technique

The same surgeon (CFVB) performed all surgical procedures employing the standard stop & chop phacoemulsification technique under topical anesthesia. 2.2 mm clear corneal incisions and 5.0 to 5.5 mm manually created capsulorhexes were employed for all surgeries, using the same ophthalmic viscosurgical device (OVD) Duovisc® (3.0% sodium hyaluronate, 4.0% chondroitin sulfate with 1.0% sodium hyaluronate ALCON Laboratories, Forth Worth TX, USA). After cataract removal and cortical material aspiration, all patients had in-the-bag implantation of either an AT LISA tri 839MP or an Acrysof IQ PanOptix in concordance to randomization. Finally, all remaining OVD under the IOL were removed.

### 2.7. Statistical Analysis

Descriptive data are shown as mean ± SD and range. Significance was assessed using the t-student and Mann–Whitney tests. The Pearson correlation coefficient (*r*) or the Spearman tests were employed according to data distribution [12]. Also, linear regression analyses were performed between angle *κ* and HOAs measurements for both presbyopia-correcting IOLs. *P* values < 0.05 were considered to be statistically significant. Gaussian distribution was determined using the D'Agostino–Pearson omnibus normality test for all variables. Statistical analyses were performed using the Statistical Package for Social Sciences (SPSS) software (version 15, SPSS, Inc., Chicago, IL; USA). Plots and layouts were composed using the Prism GraphPad software (Prism Inc., version 8.0).

## 3. Results

The study comprised a total of 43 eyes from 43 patients: twenty-three eyes in the Acrysof IQ PanOptix group and twenty patients in AT LISA tri 839MP group. An in-the-bag IOL positioning was achieved in all cases.

### 3.1. Preoperative Measurements

No statistically significant differences were evidenced for age and gender between groups. Preoperative data of included patients are summarized in Table 2.

### 3.2. Postoperative Measurements

Six months after the surgical procedure UDVA, UNVA, UIVA, and *κ* distance measurements were evaluated. Mean postoperative visual acuity for all distances and angle *κ* distance measurements at the six-month follow-up visit are shown in Table 3.

Total HOAs and internal aberrations were evaluated at the six-month follow-up visit. No statistically significant differences were evidenced between groups, as depicted in Table 4.

The Pearson correlation coefficient (*r*) and linear regression analyses were obtained between angle *κ* distance and UDVA, UNVA, and UIVA. A nonsignificant mild inverse correlation was assessed, as shown in Table 5.

Also, the Pearson correlation coefficient (*r*) was obtained between angle *κ* distance and total HOAs and internal aberrations. A mild nonsignificant positive correlation was evidenced as depicted in Table 6.

In order to assess visual quality parameters, we obtained the correlation coefficient (*r*) between total HOAs and the Strehl ratio, finding a statistically significant inverse correlation for both IOLs.

Three patients were withdrawn from the final study analysis due to one surgical complication (zonular dehiscence), and two patients failed to attend to their scheduled appointments after surgery.

## 4. Discussion

Implantation of multifocal IOLs has been associated with reduced image quality and undesirable visual phenomena [13–15]. Studies have shown that multifocal IOLs are associated with a higher incidence of optical aberrations, causing more halos and glare, than other types of IOLs [17–20].

There are few studies on the influence of the angle *κ* on the visual quality of trifocal IOLs [21]. Qi et al. recently reported that the size of angle *κ* affected the incidence of glare and halo after trifocal IOL implantation, but that there were no significant effects on the postoperative vision. The impact on objective visual quality varied depending on the patient groupings used; these results might have been attributable to the small sample size or short follow-up time [21].

Consequently, among other factors, including the pupil size and the magnitude of preoperative astigmatism, the angle *κ* is to be considered when analyzing a potential trifocal IOL candidate [3]. Also, several studies have suggested that both the higher-order aberrations and the angle *κ* play a vital role in predicting the postoperative satisfaction after implanting a multifocal IOL [4–7].

Harrer et al. reported high variability in angle *κ* measurement in a large number of pseudophakic patients associated with age and axial length. However, in a regression model including all cases, the effect of axial length on the angle *κ* was weak due to the limited number of hyperopic eyes.

Moreover, HOAs were generally correlated weakly with the amount of angle *κ*. Nonetheless, a significant correlation was observed for astigmatism of the 4th order [4].

In our study, the mean postoperative visual acuity was optimal for distance, intermediate, and near vision in both groups; which confirms that both trifocal IOLs can provide good postoperative outcomes. However, no significant correlation was evidenced between the postoperative visual acuity and angle *κ* distance for any trifocal IOL. These findings suggest that the influence of moderate angle *κ* distance (mean angle *κ* distance of 0.337 ± 0.15, range 0.10–0.62; and 0.278 ± 0.13 range 0.02–0.64, for each group, respectively) has no significant effect on the visual acuity after trifocal IOL implantation. Similarly, no significant correlation was found between higher-order aberrations, both internal and total aberrations, and the angle *κ* distance for both trifocal IOLs; which further indicates that there is no significant association between these variables. Previous reports by Basmak et al. have described a significant correlation between positive refractive errors and large positive angle *κ* values [20]. However, these findings are evident when a considerable number of patients depict large positive angle *κ* measurements and positive refractive errors.

It is essential to bear in mind that the inner optical diameter of each trifocal intraocular lens is slightly different. The PanOptix inner diameter is 1.164 mm, while the AT LISA tri is 1.04 mm [13]. This particular feature allows the former a larger angle *κ* of 0.58 mm without associated visual phenomena according to the manufacturer when compared with the latter, with a suggested maximum *κ* angle of 0.52 mm. Nevertheless, for the included population, this factor seemed to have no influence regardless of the preoperative angle *κ* measurement on postoperative visual acuity for any distance.

On the other hand, a statistically significant inverse correlation was found between total higher-order aberrations and the Strehl ratio, which indicates the more the decisive decrease on the Strehl ratio, the more HOAs we encounter, with the consequent decrement on vision quality.

The Strehl ratio is the quotient of the peak intensity of an aberrated point spread function (PSF) to the ideal diffraction-limited PSF, with a value of 1.0 signifying perfect optical quality [20]. Moreover, the corneal Strehl ratio indicates the level of image quality in the presence of wavefront aberrations and provides one of the highest correlations with a visual performance. Our findings are in concordance to previously described data on the Strehl ratio and HOAs correlation [16–20].

Previously reported data have described that the size of the angle *κ* affected the visual quality of patients after trifocal IOL implantations [17]; specifically, when the angle *κ* distance was greater than 0.5 mm, patients' visual quality decreased, and when the angle *κ* was more significant than 0.4 mm, the incidence of glare and halo increased. However, in our study, no significant effects were evident in the postoperative vision, regardless of angle *κ* for both trifocal IOLs.

Another critical aspect of our study is that we yielded the angle *κ* distance in millimeters using the OPD-Scan III analyzer. The concept of an angle exists primarily in theoretical eye models and ray tracing. Clinically, the concept of displacement or a chord length is more relevant [17]. While some anterior segment imaging devices, like the OPD-Scan III (NIDEK Co., Ltd., Tokyo, Japan) report “angle” kappa, they are in fact reporting a 2-dimensional Cartesian displacement that roughly correlates with the concept of angle *κ*. The use of the term “chord” instead of “angle” emphasizes the entity described, as well as its uniqueness in the literature, and the letter “mu” replaces previously used terms with historically conflicting or misused definitions [17]. Since the pupil center can shift with miosis and mydriasis, the description of chord mu should optionally include the state of the pupil [17, 22]. Current optical biometers and topographers report chord length *κ* (an approximation of angle *κ*). The Galilei anterior segment analysis system (Ziemer Ophthalmic Systems) displays *X*-*Y* Cartesian coordinates between the corneal vertex and pupil center; the distance between the corneal vertex and the pupil center (*X* and *Y* Cartesian values) can then be used to estimate the angle *κ* [17].

Several limitations of this study should be considered. Only objective measurements of visual outcomes were obtained, without taking into consideration the individual subjective patients' perception. Another limitation is that the number of patients with large angle *κ* distance is limited, and therefore, more cases are needed to support these findings further. Also, the sample size is not sufficient enough to provide information conducive to regulate conduct in this regard. Finally, no preoperative HOAs were measured; which could give a distinctive perspective to the previous state of the patient.

In summary, patients of both groups demonstrated excellent visual performance. No significant correlation was evidenced between the postoperative visual acuity and angle *κ* distance for both groups. These findings suggest that the influence of angle *κ* has no significant effect on the visual acuity when using these trifocal IOLs. Further in vivo studies of a population with different preoperative corneal aberrometry profiles would provide insight into the influence of higher-order aberrations on trifocal intraocular lenses.

## 5. Conclusion

In our study, both trifocal IOLs showed excellent postoperative visual performance at all distances at the six-month follow-up visit. Moreover, no significant association was found between angle *κ* distance and postoperative visual acuity regardless of the angle *κ* magnitude and inner optical diameter for the two trifocal IOL included.

## Figures and Tables

**Table 1:** Trifocal IOL features [10].

Feature	Acrysof IQ PanOptix®	AT LISA tri 839MP®
Technology	Trifocal	Trifocal
Diffractive zone (mm)	4.5	6.0
Central zone	Diffractive	Diffractive
Optic type	Nonapodized	Nonapodized
Optic diameter (mm)	6.0/4.5 mm diffractive region	6.0/4.3 mm trifocal/4.3 to 6.0 mm bifocal
Near add power (D)	+3.25	+3.33
Intermediate IOL power (D)	+2.17	+1.66
Asphericity (*μ*m)	−0.10	−0.18
IOL color	Yellow	Clear
Inner ring optical diameter	1.164 mm	1.04 mm

**Table 2:** Preoperative measurements.

Parameter	Value	Acrysof IQ PanOptix	AT LISA tri 839MP	Difference between means	*P* value^*∗*^
Age	Mean ± SD	67.52 ± 8.22	65.29 ± 8.12	2.23 ± 2.7	0.423
	Range	57–81	52–80		
UDVA (logMAR)	Mean ± SD	0.324 ± 0.38	0.360 ± 0.42	0.03 ± 0.02	0.251
	Range	0.09–2.00	1.1–2.00		
Spherical equivalent (D)	Mean ± SD	0.24 ± 2.1	0.34 ± 3.2	0.01 ± 0.01	0.224
	Range	−6.25 to +4.50	−7.50 to +3.50		
Corneal astigmatism (D)	Mean ± SD	0.43 ± 031	0.51 ± 0.23	0.04 ± 0.10	0.683
	Range	0–1.00	0.25–0.1.00		
Steep keratometry (D)	Mean ± SD	44.15 ± 1.30	43.80 ± 1.29	0.36 ± 0.70	0.210
	Range	41.7–46.5	42.0–46.3		
Flat keratometry (D)	Mean ± SD	43.50 ± 1.23	43.20 ± 1.47	0.39 ± 0.63	0.152
	Range	40.0–45.3	41.2–45.1		

UDVA = uncorrected distance visual acuity. ^*∗*^Mann–Whitney test.

**Table 3:** Mean visual acuity and angle *κ* values per group at the six-month follow-up visit.

Parameter	Value	Acrysof IQ PanOptix	AT LISA tri 839MP	95% CI	*P* value^*∗*^
UDVA (logMAR)	Mean ± (SD)	0.092 ± 0.10	0.050 ± 0.06	0.04, 0.13	0.229
	Range	0–0.39	0–0.27		
UIVA (logMAR)	Mean ± (SD)	0.173 ± 0.18	0.182 ± 0.11	0.08, 0.13	0.669
	Range	0–0.91	0–0.39		
UNVA (logMAR)	Mean ± (SD)	0.068 ± 0.04	0.085 ± 0.07	0.08, 0.17	0.221
	Range	0–0.09	0–0.30		
Angle *κ* distance (mm)	Mean ± (SD)	0.337 ± 0.15	0.278 ± 0.13	−0.24, 0.11	0.093
	Range	0.10–0.62	0.02–0.64		

UDVA = uncorrected distance visual acuity; UIVA = uncorrected intermediate visual acuity; UNVA = uncorrected near visual acuity. ^*∗*^Mann–Whitney test.

**Table 4:** Comparison of the postoperative total and internal aberrations per group.

	Value	Acrysof IQ PanOptix	AT LISA tri 839MP	Difference between medians	*P* value^*∗*^
Total aberrations					
TILT (*μ*m)	Mean ± SD	0.291 ± 0.22	0.467 ± 0.45	0.004	0.387
	Range	0.01–1.05	0.07–1.54		
HOAs (*μ*m)	Mean ± SD	0.381 ± 0.21	0.485 ± 0.26	0.111	0.195
	Range	0.08–0.86	0.10–1.00		
COMA (*μ*m)	Mean ± SD	0.133 ± 0.11	0.247 ± 0.23	0.031	0.164
	Range	0.02–0.58	0.05–0.65		
TREFOIL (*μ*m)	Mean ± SD	0.289 ± 0.18	0.255 ± 0.56	0.081	0.073
	Range	0.02–0.76	0.15–2.17		
SPHERE (*μ*m)	Mean ± SD	0.045 ± 0.04	0.326 ± 0.40	0.098	0.075
	Range	0.00–0.17	0.00–1.00		

Internal aberration					
TILT (*μ*m)	Mean ± SD	0.440 ± 0.39	1.11 ± 2.18	0.015	0.401
	Range	0.05–1.50	0.07–9.30		
HOAs (*μ*m)	Mean ± SD	0.435 ± 0.67	0.831 ± 1.16	0.547	0.065
	Range	0.10–3.46	0.17–4.0		
COMA (*μ*m)	Mean ± SD	0.183 ± 0.20	0.443 ± 0.42	0.086	0.071
	Range	0.01–0.97	0.05–1.2		
TREFOIL (*μ*m)	Mean ± SD	0.289 ± 0.18	0.653 ± 1.15	0.054	0.256
	Range	0.05–2.13	0.15–2.17		
SPHERE (*μ*m)	Mean ± SD	0.140 ± 0.12	0.524 ± 0.65	0.019	0.509
	Range	0.02–0.57	0.00–2.29		

^*∗*^Mann–Whitney test.

**Table 5:** Correlation between angle *κ* distance and UDVA, UNVA, and UIVA.

	*r*	95% CI	*R* ^2^	*P* value^*∗*^
Acrysof IQ PanOptix (*n* = 23)				
UDVA (logMAR)	−0.127	−0.52, −0.31	0.016	0.573
UIVA (logMAR)	−0.279	−0.62, 0.16	0.077	0.208
UNVA (logMAR)	−0.095	−0.49, 0.33	−0.009	0.671
AT LISA tri 839MP (*n* = 23)				
UDVA (logMAR)	−0.432	−0.39, 0.87	0.187	0.284
UIVA (logMAR)	−0.360	−0.84, 0.46	0.130	0.380
UNVA (logMAR)	−0.452	−0.87, 0.36	−0.206	0.258

UDVA = uncorrected distance visual acuity; UNVA = uncorrected near visual acuity; UIVA = uncorrected intermediate visual acuity. ^*∗*^Pearson correlation coefficient (*r*).

**Table 6:** The correlation coefficient (*r*) between angle *κ* distance and internal aberration.

	*r*	95% CI	*R* ^2^	*P* value^*∗*^
Acrysof IQ PanOptix				
Total HOAs (D)	0.371	−0.05, 0.68	0.138	0.088
Internal aberration (D)	0.304	−0.13, 0.64	0.092	0.168
AT LISA tri 839MP				
Total HOAs (D)	0.173	−0.27, 0.56	0.030	0.226
Internal aberration (D)	0.240	−0.21, 0.60	0.57	0.146

^*∗*^Pearson correlation coefficient (*r*).

## Data Availability

The data used to support the findings of this study are available from the corresponding author upon request.

## References

[1] Karhanová M., Pluháček F., Mlčák P., Vláčil O., Šín M., Marešová K. (2015). The importance of angle kappa evaluation for implantation of diffractive multifocal intra-ocular lenses using pseudophakic eye model. *Acta Ophthalmologica*.

[2] Velasco-Barona C., Cervantes-Coste G., Mendoza-Schuster E. (2018). Comparison of biometric measurements obtained by the verion image-guided system versus the auto-refracto-keratometer. *International Ophthalmology*.

[3] Braga-Mele R., Chang D., Dewey S. (2014). Multifocal intraocular lenses: relative indications and contraindications for implantation. *Journal of Cataract & Refractive Surgery*.

[4] Harrer A., Hirnschall N., Tabernero J. (2017). Variability in angle *κ* and its influence on higher-order aberrations in pseudophakic eyes. *Journal of Cataract & Refractive Surgery*.

[5] Prakash G., Agarwal A., Prakash D. R., Kumar D. A., Agarwal A., Jacob S. (2011). Role of angle kappa in patient dissatisfaction with refractive-design multifocal intraocular lenses. *Journal of Cataract & Refractive Surgery*.

[6] Artal P., Berrio E., Guirao A., Piers P. (2002). Contribution of the cornea and internal surfaces to the change of ocular aberrations with age. *Journal of the Optical Society of America A*.

[7] Hashemi H., KhabazKhoob M., Yazdani K., Mehravaran S., Jafarzadehpur E., Fotouhi A. (2010). Distribution of angle kappa measurements with orbscan II in a population-based survey. *Journal of Refractive Surgery*.

[8] Choi S. R., Kim U. S. (2013). The correlation between angle kappa and ocular biometry in Koreans. *Korean Journal of Ophthalmology*.

[9] Chylack L. T., Wolfe J. K., Singer D. M. (1993). The lens opacities classification system III. *Archives of Ophthalmology*.

[10] Saucedo-Urdapilleta R., González-Godínez S., Mayorquín-Ruiz M., Moragrega-Adame E., Velasco-Barona C., González-Salinas R. (2019). Comparative analysis and repeatability assessment of IOL Master 500 versus IOL Master 700 biometry in cataract patients. *Revista Mexicana de Oftalmología*.

[11] He W., Qiu X., Zhang S. (2018). Comparison of long-term decentration and tilt in two types of multifocal intraocular lenses with OPD-Scan III aberrometer. *Eye*.

[12] de Winter J. C. F., Gosling S. D., Potter J. (2016). Comparing the Pearson and Spearman correlation coefficients across distributions and sample sizes: a tutorial using simulations and empirical data. *Psychological Methods*.

[13] Carson D., Xu Z., Alexander E., Choi M., Zhao Z., Hong X. (2016). Optical bench performance of 3 trifocal intraocular lenses. *Journal of Cataract & Refractive Surgery*.

[14] Miháltz K., Knorz M. C., Alió J. L. (2011). Internal aberrations and optical quality after femtosecond laser anterior capsulotomy in cataract surgery. *Journal of Refractive Surgery*.

[15] Yamauchi T., Tabuchi H., Takase K., Ohsugi H., Ohara Z., Kiuchi Y. (2013). Comparison of visual performance of multifocal intraocular lenses with same material monofocal intraocular lenses. *PLoS One*.

[16] Mahajan V. N. (1983). Strehl ratio for primary aberrations in terms of their aberration variance. *Journal of the Optical Society of America*.

[17] Park C. Y., Oh S. Y., Chuck R. S. (2012). Measurement of angle kappa and centration in refractive surgery. *Current Opinion in Ophthalmology*.

[18] Pérez-Merino P., Marcos S. (2018). Effect of intraocular lens decentration on image quality tested in a custom model eye. *Journal of Cataract & Refractive Surgery*.

[19] Lee J. A., Song W. K., Kim J. Y., Kim M. J., Tchah H. (2019). Femtosecond laser-assisted cataract surgery versus conventional phacoemulsification: refractive and aberrometric outcomes with a diffractive multifocal intraocular lens. *Journal of Cataract & Refractive Surgery*.

[20] Basmak H., Sahin A., Yildirim N., Papakostas T. D., John Kanellopoulos A. (2007). Measurement of angle kappa with synoptophore and orbscan II in a normal population. *Journal of Refractive Surgery*.

[21] Qi Y., Lin J., Leng L. (2018). Role of angle *κ* in visual quality in patients with a trifocal diffractive intraocular lens. *Journal of Cataract & Refractive Surgery*.

[22] Chang D. H., Waring G. O. (2014). The subject-fixated coaxially sighted corneal light reflex: a clinical marker for centration of refractive treatments and devices. *American Journal of Ophthalmology*.

